# Use of ECT in Major Vascular Neurocognitive Disorder with Treatment-Resistant Behavioral Disturbance following an Acute Stroke in a Young Patient

**DOI:** 10.1155/2019/9694765

**Published:** 2019-04-21

**Authors:** Kyle E. Rodenbach, Daniel Varon, Timothey Denko, Ryan Peterson, Carmen Andreescu

**Affiliations:** University of Pittsburgh Medical Center, Department of Psychiatry, 3811 O'Hara Street, Pittsburgh, PA 15213, USA

## Abstract

The following case describes the utilization of bitemporal ECT as a treatment of last resort in a 47-year-old woman with profoundly treatment-resistant behavioral disturbance poststroke. The use of ECT led to improvement in symptoms sufficient for discharge from an inpatient psychiatric unit to the nursing home. Neuropsychiatric sequelae of stroke include poststroke depression, anxiety, mania, psychosis, apathy, pathological laughter and crying, catastrophic reaction, and mild and major vascular neurocognitive disorders. Behavioral disturbance is common and may pose diagnostic and therapeutic difficulty in the poststroke patient. In most cases, first-line treatment includes pharmacologic intervention tailored to the most likely underlying syndrome. Frequent use of sedating medications is a more drastic option when behaviors prove recalcitrant to first-line approaches and markedly affect quality of life and patient safety. ECT is generally safe, is well tolerated, and may be effective in improving symptoms in treatment-resistant behavioral disturbance secondary to stroke with major neurocognitive impairment, as suggested in this case.

## 1. Introduction

With national and global rates of 795,000 and 15 million events per year, respectively, stroke remains a common and serious neurologic disorder with numerous well described neuropsychiatric sequelae. Poststroke depression, anxiety, mania, and psychosis have been documented in the literature, as have other neuropsychiatric syndromes including pathological laughter and crying (PLAC), poststroke apathy, and the catastrophic reaction [1]. When multiple cognitive domains are affected, patients may meet full criteria for mild or major vascular neurocognitive disorder (dementia) with or without behavioral disturbance [2].

With various presentations depending on the acuity, number, and locations of lesions, vascular dementia is a heterogeneous clinical entity. Presentation can therefore be acute or insidious and progression may range from static to step-wise. Pure vascular causes account for between 10 and 20 percent of dementia cases and are more commonly comorbid with Alzheimer's pathology [1]. Poststroke delirium is also common and must be identified and addressed prior to consideration of other neuropsychiatric sequelae to avoid misdiagnosis [3].

While treatment for poststroke depression is relatively well-established, treatments for poststroke anxiety, mania, psychosis, apathy, PLAC, and the catastrophic reaction have been understudied [4]. Pharmacotherapies for poststroke syndromes may include antidepressants, mood stabilizers, anticonvulsants, antipsychotics, or stimulants depending on the constellation of symptoms that are present [5]. When mild or major neurocognitive disorder is present, the mainstay of treatment is medical therapy targeted at vascular risk factors such as hypertension [6]. There is no evidence to suggest that cognitive enhancers (cholinergic agonists) are useful in vascular dementia [1]. Beta adrenergic antagonists may reduce agitation in patients with brain injury; however, evidence in stroke patients is limited [7].

Behavioral disturbance is common to dementia of all types. Approximately 70% of individuals with dementia experience agitation and 75% experience symptoms of psychosis [8]. Treatment of behavioral disturbances (agitation, aggression, aberrant vocalization, and interference/refusal of care) is a common reason for admission to the geriatric psychiatric unit and frequently involves careful consideration of the risks and benefits associated with pharmacologic treatment of these symptoms, particularly in the era of FDA black box warnings suggesting increased risk of mortality in elderly individuals with dementia treated with antipsychotics. While there is no FDA-approved treatment for behavioral disturbance in dementia, various classes of medications are commonly used depending on target symptoms, including antidepressants, atypical antipsychotics, anticonvulsants, and benzodiazepines [9]. Treatment strategies for behavioral disturbance resistant to traditional nonpharmacologic and pharmacologic management are limited. A recent review found that up to 88% of individuals with dementia with behavioral disturbance have favorable responses to ECT with limited and transient adverse effects associated with ECT treatments [10].

## 2. Case Report

**Figure 1:**
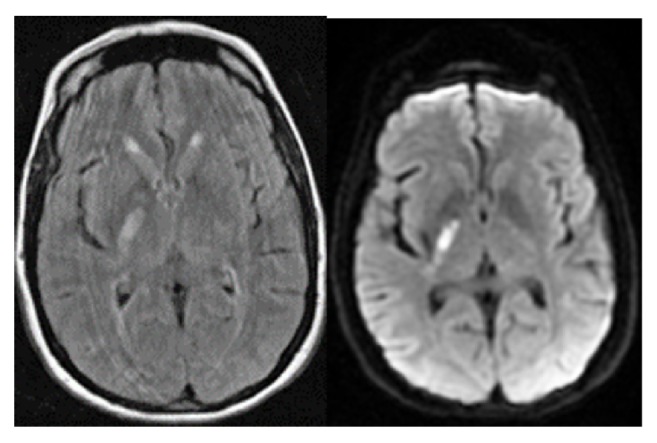
T2 FLAIR and DWI on initial presentation.

The patient is a 47-year-old Caucasian female who presented to the Emergency Department of an academic tertiary-care hospital in the Midwestern United States with complaint of left-sided weakness of the upper and lower extremities and right gaze preference three weeks after a right pontomedullary infarct complicated by Posterior Reversible Encephalopathy Syndrome (PRES) [that initial infarct had been treated in a different state]. Imaging revealed an acute infarct in the posterior limb of the right internal capsule without hemorrhagic transformation and an acute punctate infarct in the right parietal subcortical white matter with corresponding diffusion restrictions, as well as remote evidence of subcortical chronic diffuse microhemorrhages (Figure 1). The Psychiatry Consultation & Liaison service was consulted on hospital day 2 after the patient reported, “I want to strangle myself with my oxygen cord.”

On initial evaluation, the patient reported history of anxiety treated previously by her primary care physician (PCP). She reported she had been frustrated with her medical condition but really did not intend to harm herself. She reported fluctuating mood since her initial stroke and had “good days and bad days.” She denied prior history of inpatient or outpatient psychiatric care or prior suicide attempts. She was oriented to person and place, but not time, was able to state the days of the week forwards, but not backwards, and endorsed visual hallucinations during her hospitalization. This presentation was felt to be consistent with delirium, and she was started on quetiapine 25 mg.

Following a six-day medical admission, the patient was discharged to the acute inpatient rehabilitation unit housed within the hospital. Extensive diagnostic studies did not reveal an underlying etiology for the strokes, which were thought to be due to uncontrolled hypertension.

Psychiatry was reconsulted by the rehab physicians for management of problematic behaviors. The patient exhibited ego-dystonic behaviors for which she would later apologize including repeatedly climbing out of bed, shouting for nursing assistance without clear need for help, shoving her fist into her mouth to induce vomiting, and periodic, purposeless screaming. These behaviors were disruptive to staff and other patients on the unit. While initially conceptualized as residual hyperactive delirium, her behaviors persisted and continued testing for underlying causes of delirium including electrolyte derangement, occult infection, new or evolving cerebrovascular event, or excess medication burden which were unrevealing

After 60 days of acute rehab, she had reached maximal benefit of that intervention and continued exhibiting behaviors incompatible with nursing home disposition. The patient was then transferred to the university's geriatric psychiatry inpatient unit on an involuntary mental health commitment for behavioral management.

Ineffective medication trials prior to transfer included quetiapine (25 mg at bedtime and 25 mg several times daily as needed), mirtazapine (7.5 mg at bedtime), olanzapine (initial trial of 2.5 mg at bedtime and 2.5 mg several times daily as needed and a second trial of 15 mg and 2.5 mg several times daily as needed), buspirone (15 mg TID), divalproex (initial trial of 750 mg at bedtime and a second trial of 500 mg TID with lactulose and levocarnitine for hyperammonemia), melatonin (9 mg at bedtime), propranolol (40 mg QID), trazodone (150 mg at bedtime), gabapentin (200 mg several times daily as needed), dextromethorphan (20 mg BID, given as Robitussin), and clonazepam (0.5 mg AM and 1 mg PM).

Throughout this period, the patient remained intermittently apologetic for her behaviors. Orientation was typically attuned to person, sometimes place, and generally not to month or year. She consistently denied depressed mood, anxiety, visual hallucinations, auditory hallucinations, paranoia, suicidal ideation, or homicidal ideation. Thought process remained concrete and perseverative with limited spontaneous speech output and paucity of thought content. Language remained intact without evidence of aphasia. Recent and remote memory were difficult to assess formally due to behavioral disturbance, but she had difficulty remembering recent details of her hospital course and remote details of her life prior to moving to her current city. She required staff assistance for completion of toileting, dressing, and feeding. She had deficiencies in executing complex motor tasks, such as getting out of bed, and was frequently found diagonal in bed with a limb tossed over the side-rail. These deficiencies were in excess of the residual motor effects of her strokes and suggestive of alterations in visuospatial skills, executive function, and planning. Her aberrant vocalizations did not appear goal-oriented and were not ameliorated by staff presence. This presentation persisted and was thought to represent a new cognitive baseline meeting diagnostic criteria for major vascular neurocognitive disorder with behavioral disturbance.

Nonpharmacological strategies including music, sensory stimulation, one to one time with staff, and frequent repositioning were tried without improvement in her symptoms. Additional ineffective medication trials following transfer to inpatient psych included fluoxetine (60 mg per day), retrial of dextromethorphan with fluoxetine as an enzymatic inhibitor (again to 20 mg BID), retrial of quetiapine (up to 600 mg total per day), haloperidol (5 mg several times daily as needed IM), oxycodone (5 QID), lorazepam (up to 6 mg daily), carbamazepine (200 TID), and chlorpromazine (50 QID). Throughout these trials, the patient continued to exhibit frequent periods of severe psychomotor agitation requiring vest restraint and purposeless screaming alternating with periods of oversedation following medications. Other than providing intermittent sedation, no particular combination of medications proved effective in treating the target symptoms.

At this point, having exhausted all reasonable behavioral and pharmacologic options, the inpatient psychiatric team recommended ECT as a last intervention prior to pursuing a palliative approach. Medical Ethics was consulted and felt ECT to be consistent with her previously articulated beliefs and wishes.

The patient was formally evaluated by the ECT service and, given her incapacity to consent, a court order was obtained for the procedure. She underwent an acute course of bitemporal ECT using a MECTA Spectrum 5000Q machine. She received methohexital and succinylcholine as anesthetic and relaxant agent, respectively. A dose-titration method was used to determine stimulus intensity. She received treatments at 50% over seizure threshold with the following parameters: pulse width: 1 millisecond, frequency: 20 Hz, duration: 2 sec. Treatments were given three times per week. She was maintained on chlorpromazine (50 mg QID) and lorazepam (1 mg QID) during the treatments. Following the sixth ECT treatment, the patient rarely engaged in purposeless yelling, and remained quiet most of the day, experienced normalization of her sleep wake cycle, but still exhibited purposeless movements and psychomotor agitation requiring a vest restraint at night.

Following the third week of ECT treatments, she was consistently having low scores on the Pittsburgh Agitation Scale (PAS) and had minimal requirements for as needed medications for agitation [11]. While she still required a vest restraint overnight, her psychomotor agitation had improved dramatically. She resumed feeding herself with her right arm and tolerated pureed foods for the first time in six months. Following an acute course of 16 treatments, ECT was tapered to twice weekly and she started sertraline 25 mg in preparation for further decrease in ECT frequency. She remained stable and was successfully discharged to a nursing home with continuation of ECT as an outpatient. Following the expiration of the original court order for ECT, outpatient ECT was discontinued and the patient's family chose to not pursue a renewal of the order for continued treatment. She received 29 treatments in total. Nursing home staff reported that her behaviors remained in control after stopping ECT and she was thereafter able to return home with her parents.

## 3. Discussion

This is a case of treatment-resistant behavioral disturbance in major neurocognitive disorder due to vascular disease in a middle-aged adult during the acute poststroke period (< 30 days). The behavioral disturbance was responsive to ECT and permitted discharge from an inpatient psychiatric unit to the nursing home. To our knowledge, there is no previously published data indicating that ECT is an effective treatment for behavioral disturbance in vascular dementia in nonelderly individuals or in the acute poststroke setting in individuals of any age.

Data on ECT in dementia with behavioral disturbance comes primarily from case reports, case series, and retrospective chart reviews, as there have been no randomized controlled trials conducted to date. The available data are mostly positive but tend to derive from older individuals with Alzheimer's disease [10].

One retrospective chart review of sixteen individuals with dementia, two due to vascular disease, with mean age of sixty-six, showed reduced scores on objective measurements of agitation, including the Pittsburgh Agitation Scale, following an acute course of ECT [11, 12]. Similarly, a prospective cohort study of twenty-three individuals with dementia with behavioral disturbance, four due to vascular disease, with mean age seventy-four found statistically significant reductions in agitation during acute ECT treatment [10]. Neither study analyzed individuals with vascular dementia separately to verify efficacy in this specific subset.

One case report of ECT in a seventy-eight-year-old woman with vascular dementia with behavioral disturbance showed improvement in treatment-refractory agitation; however, the course of this individual followed the classic, step-wise progression of vascular dementia with gradual accumulation of subcortical white matter disease rather than that of an acute and significant infarct as in this case [13]. One case report describes successful use of ECT for agitation in a relatively young (fifty-seven-year-old) patient with early-onset Alzheimer's disease [14].

Additionally, data regarding appropriate use of maintenance ECT for agitation in dementia are extremely limited. One case report suggests that alternating periods of acute and maintenance frequencies driven by symptom burden may be effective in managing agitation and that slow tapering over weeks to months may be necessary [15].

While the available data suggest ECT is effective in treatment-resistant agitation in dementia, it is uncommonly used for this purpose, and this practice has not yet been rigorously evaluated in randomized controlled trials. Little is known regarding the efficacy of ECT in management of behavioral symptoms in individuals with vascular dementia. Moreover, due to the heterogeneity of vascular dementia as a diagnostic entity, future studies should aim to recruit individuals with a variety of clinical presentations across the lifespan, as individuals with acute infarcts may respond differently than individuals with a step-wise pattern of progression. Additional information is also needed regarding appropriate tapering schedules and maintenance regimens following acute ECT for this indication.

Treatment-resistant behavioral symptoms in dementia can be extremely distressing to families, caregivers, and patients. ECT may be appropriate for select cases.
